# Transpiration efficiency: insights from comparisons of C_4_ cereal species

**DOI:** 10.1093/jxb/erab251

**Published:** 2021-06-03

**Authors:** Vincent Vadez, Sunita Choudhary, Jana Kholová, C Tom Hash, Rakesh Srivastava, A Ashok Kumar, Anand Prandavada, Mukkera Anjaiah

**Affiliations:** 1 Institut de Recherche pour le Développement (IRD), UMR DIADE, University of Montpellier, Montpellier, France; 2 International Crops Research Institute for the Semi-Arid Tropics (ICRISAT), Patancheru, Telangana, India; 3 Multi-Crop Research Centre, Corteva Agriscience, Agriculture Division of Dow-DuPont, Tunki-kalsa, Wargal Mandal, Siddipet, Telangana State, India; 4 The University of Queensland, Australia

**Keywords:** Aquaporin, breeding, climate change, drought, maize, pearl millet, *Pennisetum glaucum*, rhizosphere, root hydraulics, soil hydraulics, *Sorghum bicolor*, *Zea mays*

## Abstract

We have previously reported that there is a tight link between high transpiration efficiency (TE; shoot biomass per unit water transpired) and restriction of transpiration under high vapor pressure deficit (VPD). In this study, we examine other factors affecting TE among major C_4_ cereals, namely species’ differences, soil type, and source–sink relationships. We found that TE in maize (10 genotypes) was higher overall than in pearl millet (10 genotypes), and somewhat higher than in sorghum (16 genotypes). Overall, transpiration efficiency was higher in high-clay than in sandy soil under high VPD, but the effect was species-dependent with maize showing large variations in TE and yield across different soil types whilst pearl millet showed no variation in TE. This suggested that species fitness was specific to soil type. Removal of cobs drastically decreased TE in maize under high VPD, but removal of panicles did not have the same effect in pearl millet, suggesting that source–sink balance also drove variations in TE. We interpret the differences in TE between species as being accounted for by differences in the capacity to restrict transpiration under high VPD, with breeding history possibly having favored the source–sink balance in maize. This suggests that there is also scope to increase TE in pearl millet and sorghum through breeding. With regards to soil conditions, our results indicate that it appears to be critical to consider hydraulic characteristics and the root system together in order to better understand stomatal regulation and restriction of transpiration under high VPD. Finally, our results highlight the importance of sink strength in regulating transpiration/photosynthesis, and hence in influencing TE.

## Teaser

Comparisons among C_4_ maize, sorghum, and pearl millet show that soil type and source–sink balance affect transpiration efficiency under high vapor pressure deficit in a species-dependent manner.

## Introduction

Transpiration efficiency (TE; shoot biomass per unit water transpired) can be calculated as TE=0.6*C*_a_(1–*C*_i_/*C*_a_)/(*W*_i_–*W*_a_), where *C*_a_ and *C*_i_ are the ambient CO_2_ concentration and the concentration in the stomatal chamber, respectively, and *W*_a_ and *W*_i_ are the ambient vapour pressure and the vapour pressure in the stomatal chamber, respectively (Condon *et al.*, 2002). We have previously reviewed considerable evidence for the existence of a genetic term in the denominator of this equation (Vadez *et al.*, 2014), which is akin to a vapor pressure deficit (VPD) factor at the leaf level. This has long been thought of as a purely environmental term and thus out of reach for genetics. However, it is known that different genotypes across a number of species can restrict transpiration under high VPD, thus suggesting that a genetic element is involved (Vadez *et al.*, 2013b). Such genotypes have a lower mean VPD value over the course of the day, and hence a higher TE (as shown in modelling outputs in Sinclair *et al.*, 2005). Vadez *et al.* (2014) thus provided new insights into the potential causes of variation in TE, moving away from the previous focus on the *C*_i_/*C*_a_ ratio on which much previous work has concentrated.

In this study, we aim to provide further insights into this ‘new’ outlook on TE, presenting experimental evidence for other drivers of its variation, building on but also going beyond the restriction of transpiration in response to increasing VPD. The aspects that we examine are related to differences among C_4_ cereal species, soil effects, and source–sink effects. We have selected maize, sorghum, and pearl millet as these are the main C_4_ cereals, accounting for 197, 42, 33×10^6^ ha worldwide, respectively, with mean yields of 5.71, 1.42, and 0.94 ton ha^−1^, respectively (http://www.fao.org/faostat/en/#data/QC/visualize; 2018 data). Maize has been subject considerable breeding efforts over the past century to improve its productivity and expand its area of geographical adaptation. However, it is considered as relatively sensitive to several abiotic stresses such as drought, low soil fertility, and waterlogging. By contrast, pearl millet and sorghum are considered to be very hardy crops, and are tolerant to harsh growing conditions such as poor soil fertility, high air temperature, and water limitation. Although they are often the only choice of staple crops for farming communities in the semi-arid tropics, they are considered as ‘orphan’ crops and have been subject to far less breeding efforts for improvement than maize.

To date, there has been very limited work towards comparing TE across species. Meanwhile, comparing TE across conditions is very difficult, because it depends on VPD conditions in the environment, and a normalization function is required (as proposed initially by de Wit, 1958, and used for instance by Seversike *et al.*, 2014). In relation to drought, although maize is considered to be less well adapted than pearl millet and sorghum, there is actually very limited quantified evidence for this assertion (but see Muchow *et al.*, 1989*a*, 1989*b*). In relation to our current study, there are very limited data comparing TE among these species. Bierhuizen and Slatyer (1965) define TE as k/(e*–*e*), where the term e*–*e* represents the gradient of vapor pressure between the leaf and the air, and k is a constant that *a priori* only distinguishes C_3_ from C_4_ species (Tanner and Sinclair 1983; Sinclair *et al.*, 1984), and encompasses *C*_i_/*C*_a_ as defined by Condon *et al.* (2002, 2004). The definition of TE by Bierhuizen and Slatyer (1965) implies that it should be similar in maize, sorghum, and pearl millet. In our current study, we have tested this implication by measuring TE in these species under different intensities of soil water stress and under different VPD conditions.

In terms of examining soil effects, water flows through a continuum from the soil to the root to the shoot and to the atmosphere. Earlier studies and reviews of TE have hypothesized that a restriction in the movement of water in the plant, possibly in the roots (Sadok and Sinclair, 2010; Schoppach *et al.*, 2014; Choudhary and Sinclair, 2014; Vadez, 2014), could explain the restriction of transpiration under high evaporative demand. However, there has been no consideration of a possible role of the soil in this response of transpiration to increasing VPD. Soils have different hydraulic capacities (e.g. Sinclair, 2005; Roy *et al.*, 2018). The rhizosphere, i.e. the minute layer surrounding the roots and ‘connecting’ them to the soil, has received recent attention in its role in sustaining the soil–plant continuum under progressive soil drying (Carminati *et al.*, 2016, 2017, 2020; Ahmed *et al.*, 2018; Carminati and Javaux, 2020). However, there has been no work that has attempted to address a possible connection between soil hydraulic conductivity and restriction of transpiration under increasing VPD. We consider this aspect here indirectly by examining TE under different VPD conditions in plants in different soils with putative differences in hydraulic characteristics.

In terms of examining source–sink effects, as noted above, Condon *et al.* (2002) presented the formula TE=0.6*C*_a_(1–*C*_i_/*C*_a_)/(*W*_i_–*W*_a_). From this definition, TE is maximized when the ratio *C*_i_/*C*_a_ is minimized, and given that ambient CO_2_ is essentially constant in the lifetime of a crop, then *C*_i_ has to be kept low for this to be achieved. This is possible either by reducing the stomatal conductance of the leaf or by increasing its carboxylation efficiency. Here, we hypothesize that any differences in the sink capacity for carbon compounds could simply lead to differences in transpiration efficiency. Despite the close relationship between photosynthetic capacity and stomatal conductance (Wong *et al.*, 1979; Ainsworth and Bush, 2011; Kelly *et al.*, 2014), the coupling between photosynthesis and stomatal opening is far from tight (Lawson and Blatt, 2014; Lawson and Vialet-Chabrand, 2019). This could in part explain putative differences in TE among genotypes, and also between species. We have therefore measured TE where the source–sink ratio has been altered.

The objectives of this paper were: (i) to examine differences in TE among C_4_ species; (ii) to examine soil effects on TE, including consideration of a possible role for soil/rhizosphere hydraulic conductivity in determining the transpiration restriction phenotype; (iii) to examine sink effects on TE, including consideration of possible links between source–sink relationships, leaf photosynthetic rates, and TE.

## Materials and methods

### Plant material

A total of 36 genotypes were used, of which 10 were maize (*Zea mays*), 16 were sorghum (*Sorghum bicolor*), and 10 were pearl millet (*Pennisetum glaucum*; see Table 1 for brief descriptions). The maize genotypes were hybrids and included seven breeding material from the Pioneer breeding pipeline for South Asia, plus a released Pioneer hybrid and two checks (Monsanto 900M Gold and ‘Public Check’). The sorghum genotypes were all breeding lines, with some released as cultivars by ICRISAT and the State Agricultural Universities in India. The pearl millet genotypes were elite single-cross hybrids with 843A, a downy mildew-resistant male-sterile line, as the female parent and the pollinators were either parents of populations or of popular hybrids in India. All the genotypes were chosen with no *a priori* knowledge of their genetic distance and while they did not represent the range of variation available in each of the species, they provided a representation of elite material that was well adapted to the geographical zone where the experiments were carried out.

**Table 1.** Description of the maize, sorghum, and pearl millet lines used in the experiments

Genotype	Characteristics	Drought response
**Maize** (all hybrids)		
8315622	Tall, high yield	Moderately tolerant
18270413	Short, early maturity	Tolerant
783527	Medium-tall, stable yield	Tolerant
4695575	Broad leaf, high yield, early maturity	Sensitive
22525674	Medium yield, medium maturity	tolerant
9424780	High yield, big cob, late maturity	Sensitive
14746185*	Tall, high and consistent yield, late flowering	Tolerant
30V92*	High yield, can be grown at high plant density, medium maturity	Tolerant
900M Gold*	Medium-tall, high yield, late maturity	Sensitive
Public Check	Medium-tall, high yield, late maturity	Sensitive
**Sorghum** (all breeding lines)		
296B	Rainy season maintainer line	Sensitive
BTx623	Template line for genome-wide analysis, High-coverage BAC library, low canopy temp	Sensitive
E 36-1	Non-stay-green check line	Tolerant
ICSR93024	–	
ICSV1	Post-rainy season, resistant to leaf diseases	–
ICSV700-P10	–	–
ICSV745	Dual purpose, midge-resistant	–
ICSV93046-P1	–	–
IS18551	–	–
IS9830	–	–
M 35-1	Widely adapted post-rainy season	Tolerant
N13	Stay-green donor	Tolerant
PB15220-1	Post-rainy season, high yielding	Sensitive
PB15881-3	Post-rainy season, high yielding	Sensitive
PVK 801-P23	Post-rainy season, high yielding	Sensitive
S35*	Stay green	Tolerant post-flowering
**Pearl millet** (all single-cross)		
841B	Medium-tall, medium-early flowering	Sensitive
Pusa 322	841B × PPMI 301	-
863B*	Medium-tall, medium-early flowering	Tolerant
GB8735	Medium-tall, early flowering	Drought escaper
ICMP 451-P6	Tall, long panicle bristles	Tolerant
H77/833-2*	Short, many tillers, photoperiod-sensitive early flowering, seedling tolerant to heat stress	Sensitive
ICMV-IS 92222	–	–
PT732B-P2	*d*_2_ dwarf, photoperiod-sensitive, late flowering	–
PRLT*	Medium-tall, early flowering, seedling sensitive to heat stress	Tolerant of terminal drought
Tift 238D1-P158	Late flowering	–

* Genotypes used in Exps 3 and 4 to examine the effects of soil type; for sorghum an additional two genotypes R16 and K359 were used that were not included in the other experiments.

For pearl millet, all genotypes are male pollinator on top of 843A, except for Pusa 322. All these genotypes are elite materials in their respective breeding programs. ‘–’ indicates that the information is not available.

### Plant growth conditions and experimental design

**Fig. 1.**
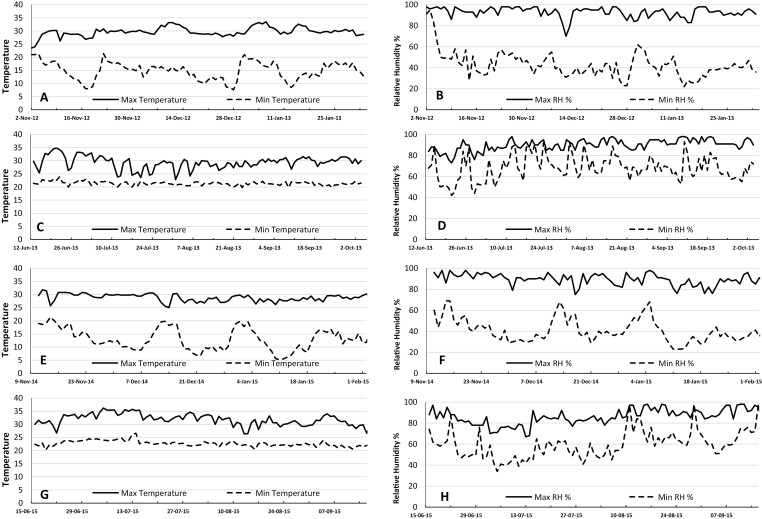
Summary of environmental conditions during the experiments. (A, C, E, G) Maximum and minimum temperatures and (B, D, F, H) maximum and minimum relative humidity in (A, B) Exps 1 and 5, (C, D) Exp. 2, (E, F) Exp. 3, and (G, H) Exp. 4.

Plants were grown individually in lysimeters, which consisted of PVC tubes of 25 cm diameter and 2.0 m length, as described by Vadez *et al.*, 2011*a*, 2013*a*). The lysimeters were placed in trenches 1.9 m deep and 1.7 m wide so that tubes were not exposed to direct radiation. The trenches were outdoors under natural conditions at the ICRISAT campus (17.32ºN, 78.21ºE), and protected by a rain-out shelter, exposing the tubes to atmospheric conditions similar to the field. The tubes were positioned to give a plant density of 10 plants m^-2^, which was similar to that used in normal field cultivation. Five experiments were carried out, two for the species comparisons under different water regimes and VPD in one soil (Alfisol) (Exps 1 and 2), two for comparisons of soil effects in four different soils and with different vapor pressure deficits (VPDs) under well-watered conditions (Exps 3 and 4), and one for comparisons of source–sink effects in one soil (Alfisol) and under well-watered conditions (Exp. 5). Experiments 1 and 5 were sown on 2 November 2012 and harvested on 2 February 2013. Experiment 2 was sown on 13 June 2013 and harvested on 4 October 2013. Experiment 3 was sown on 11 November 2014 and harvested on 5 February 2015. Experiment 4 was sown on 16 June 2015 and harvested on 16 September 2015. Experiments 1, 3 and 5 were carried out post-rainy season, typically characterized by a medium-to-high evaporative demand with mean maximum daily VPDs of 2.30–2.40 kPa. Experiments 2 and 4 were carried out in the rainy season, typically characterized by a low-to-medium mean maximum daily VPD of 1.40 kPa (Exp. 2) and 2.00 kPa (Exp. 4) (Fig. 1).

#### Comparisons across different species, water regimes, and VPDs (Exps 1 and 2)

In both experiments a single period of water stress was imposed on the plants by stopping irrigation at different points in the crop cycle. Prior to stress being imposed, the lysimeters were watered regularly to ~90% field capacity, and this regime was continued in the well-watered controls. During that phase, watering was applied each time after the lysimeters were weighed (see below), which was either every 7 d or every 14 d. An additional 2–4 L of water between weighings was applied when these were separated by 14 d (with the weight of water applied being recorded). In Exp. 1, the stress treatment in each species began at the time of its flowering (tassel emergence in maize), which corresponded to 60 d after sowing (DAS) in maize, 57 DAS in sorghum, and 44 DAS in pearl millet. In Exp. 2, three water-stress treatments were imposed by stopping irrigation for all the species at the time of flowering of each one; thus, water stress treatments began for all species at 48 DAS (pearl millet flowering), at 61 DAS (sorghum), and at 68 DAS (maize tassels). In this experiment, the last irrigation was an addition of 2 L water to each lysimeter. Experiment 1 experienced higher VPD conditions than Exp. 2 (Fig. 1).

#### Comparisons across different soils and VPDs under well-watered conditions (Exps 3 and 4)

Three genotypes were selected for each species. These were either popular cultivars or standard checks used in earlier studies and in many trials (see Table 1). Four different soils were used. The Alfisol (which was the same in Exps 1, 2, and 5) and the Vertisol were collected from the ICRISAT farm and are described in detail by Bhattacharyya *et al.* (2020). The sand:silt:clay ratios were 64:9:27 for the Alfisol and 31:19:50 for the Vertisol. A loamy sand soil was also used and consisted of sediment collected from the bottom of an empty water tank at the ICRISAT campus. The final soil was a sandy soil generated by mixing construction sand and the Alfisol in a 3:1 ratio (v/v). The lysimeters were maintained in a well-watered condition, as described above for Exps 1 and 2. Experiment 3 experienced higher VPD conditions than the rainy-season Exp. 4 (Fig. 1).

#### Comparisons of different source–sink balance under different water regimes (Exp. 5)

Different source–sink balances were obtained by severing the panicles/cobs as they were emerging. Maize cobs were removed before silking and one severing was sufficient. Sorghum and pearl millet panicles were removed at the time of booting; however, it was necessary to sever repeatedly to remove new panicles from the tillers and even nodal tillers in most of the pearl millet genotypes and in some sorghum. The cobs/panicles were oven-dried and their weight was included in the shoot biomass at harvest. The lysimeters were maintained either in a well-watered condition as described in Exps 1 and 2, or subjected to water stress that was imposed by giving the last irrigation at 59 DAS in all three species at the same time.

### Measurement of transpiration and determination of transpiration efficiency

Weighing of the lysimeters started at 27, 27, 18, 26, and 27 DAS in Exps 1 to 5, respectively, and was carried out six times in Exps 1 and 5, seven times in Exp. 2, and nine times in Exps 3 and 4. The weights were measured using a S-type load cell (Mettler-Toledo, Geneva, Switzerland), with a 200-kg capacity and precision of 20 g. In terms of the watering regime, the lysimeters were weighed immediately before the weekly irrigation was applied. Soil evaporation was prevented by covering the surfaces of the tubes before the first weighing with a plastic sheet, which in turn was covered with a 2-cm layer of plastic beads. Transpiration was determined as the difference in weight between successive weighings, allowing for any additional application of water between the weighings to be taken into account in the overall transpiration calculation. The sum of the transpiration values gave the total plant transpiration (also referred to as ‘water used’ in the figures), which was then used to calculate transpiration efficiency (TE) as the ratio of the total shoot biomass (panicles/cobs plus vegetative above-ground biomass) to the total transpiration. For the plants exposed to the water-stress treatment, the difference between the first weighing, when the tubes were at field capacity, and the last weighing after final harvest gave the total water extracted from the lysimeters, which represented a measure of the plant capacity to extract water from the soil profile under water stress. At harvest, plants were separated into leaves, stems, and panicles/cobs. For maize, the tassel was included in the stem fraction. The panicles or cobs were threshed to obtain grain weight. In Exp. 5, the weight of the severed panicles and cobs was added to the total shoot biomass. The experiments were in a complete randomized split-split block design with either water regime, soil, or panicle/cob removal as the main factor, species as the first sub-factor, and genotypes randomized within each sub-factor with five replicates. One- and two-way ANOVA were carried out to analyse genotype and genotype-by-treatment interaction effects within each species. LSD values were used to compare the means among treatments and species.

## Results

### Species differences in TE, yield, and water use under different water stress and VPD conditions

**Fig. 2.**
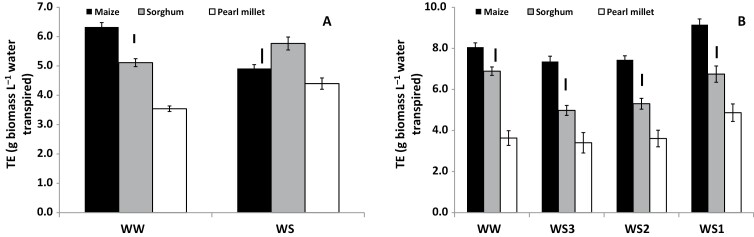
Transpiration efficiency (TE) in maize, sorghum, and pearl millet grown in the field in lysimeters under different water regimes. (A) Experiment 1 with relatively high vapor pressure deficit (VPD; Fig. 1). Plants were either well-watered (WW) or subjected to water stress (WS) by stopping watering at 60 d after sowing (DAS) in maize, 57 DAS in sorghum, and 44 DAS in pearl millet, corresponding to tassel emergence in maize, and flowering in sorghum and pearl millet. (B) Experiment 2 with relatively low VPD. In addition to the WW treatment, three different WS regimes were applied: WS1, stress imposed on all species at the time of tassel emergence in maize; WS2, stress imposed on all species at the time of flowering in sorghum; WS3, stress imposed on all species at the time of flowering in pearl millet. Transpiration efficiency (TE) was computed as the ratio of the total plant biomass (panicle, if any, plus vegetative aboveground biomass) divided by the total transpiration (from the beginning of cylinder weighing to harvest). Data are means (±SE) of 10 genotypes for maize and pearl millet, and 16 genotypes of sorghum, with five replicate plants being sampled to determine the mean for each genotype. Bars indicating the LSD at *P*<0.01 within each water treatment are shown.

**Fig. 3.**
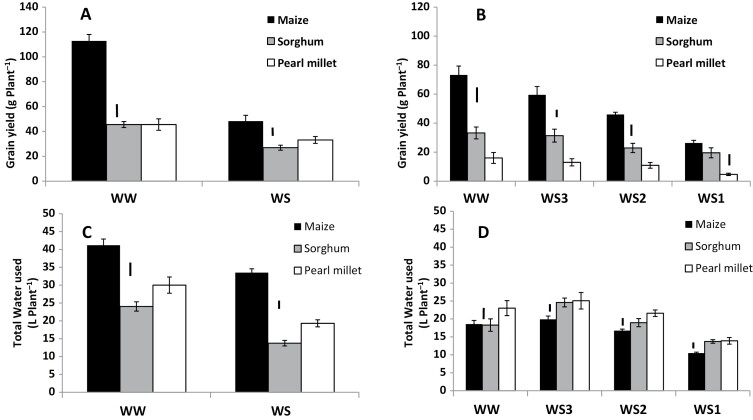
Grain yield and total water used in maize, sorghum, and pearl millet grown in the field in lysimeters under different water regimes. (A, C) Experiment 1 with relatively high vapor pressure deficit (VPD; Fig. 1). Plants were either well-watered (WW) or subjected to water stress (WS) by stopping watering at 60 d after sowing (DAS) in maize, 57 DAS in sorghum, and 44 DAS in pearl millet, corresponding to tassel emergence in maize, and flowering in sorghum and pearl millet. (B, D) Experiment 2 with relatively low VPD. In addition to the WW treatment, three different WS regimes were applied: WS1, stress imposed on all species at the time of tassel emergence in maize; WS2, stress imposed on all species at the time of flowering in sorghum; WS3, stress imposed on all species at the time of flowering in pearl millet. Data are means (±SE) of 10 genotypes for maize and pearl millet, and 16 genotypes of sorghum, with five replicate plants being sampled to determine the mean for each genotype. Bars indicating the LSD at *P*<0.01 within each water treatment are shown.

There were large differences in TE among the three species. Under well-watered (WW) conditions in both Exps 1 and 2 maize had higher TE than sorghum, which in turn had higher TE than pearl millet (Fig. 2). The same pattern was observed in the three water-stress (WS) treatments in Exp. 2 when VPD was relatively low (Fig. 2B), whereas in Exp. 1 when VPD was relatively high the value of TE in maize reduced to the same level as that in pearl millet (Fig. 2A). Yield results were generally in agreement with the TE results (Fig. 3A, B), with maize having a higher yield than sorghum and pearl millet under both WW and WS conditions regardless of the VPD. Sorghum yield was similar to that of pearl millet in the high-VPD season (Exp. 1; Fig. 3A), but it was higher in the low-VPD season, regardless of the water treatment (Exp. 2; Fig. 3B). The results for water use were somewhat different from those for TE and yield. In the high-VPD season maize used more water than sorghum and pearl millet, although not in proportion to the difference in grain yield (Fig. 3C). Interestingly, in the low-VPD season, water use was similar in all three species, and even tended to be slightly higher in pearl millet, despite its lower yield than maize (Fig. 3D). Maize extracted more water from the soil profile under WS in the high-VPD season, whilst water extraction was of the same order in all three species in the low VPD-season (Supplementary Fig. S1).

### Soil effects on TE, yield, and water use under different VPD conditions

**Fig. 4.**
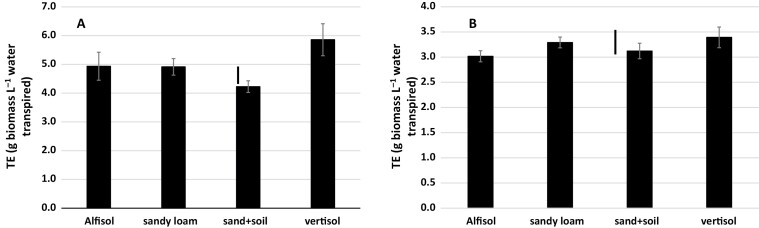
Mean transpiration efficiency (TE) across maize, sorghum, and pearl millet grown in different soil types in lysimeters in the field under well-watered conditions. (A) Experiment 3 with relatively high vapor pressure deficit (VPD) and (B) Experiment 4 with relatively low VPD (Fig. 1). Data are means (±SE) of all the species, based on the means of three genotypes in each one (Table 1), with five replicate plants being sampled to determine the mean for each genotype. Bars indicating the LSD at *P*<0.01 between the soil treatments are shown.

**Fig. 5.**
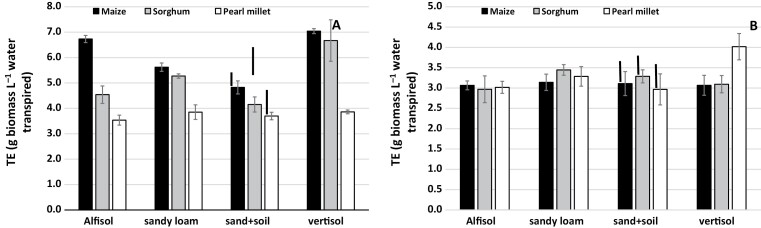
Mean transpiration efficiency (TE) for maize, sorghum, and pearl millet grown in different soil types in lysimeters in the field under well-watered conditions. (A) Experiment 3 with relatively high vapor pressure deficit (VPD) and (B) Experiment 4 with relatively low VPD (Fig. 1). Data are means (±SE) of three genotypes in each species (Table 1), with five replicate plants being sampled to determine a mean value for each genotype. Bars indicating the LSD at *P*<0.01 between the soil treatments within a species are shown. In (A) the LSD to compare differences in TE between species is 0.37 g biomass L^−1^ water transpired. In (B) the LSD to compare differences in TE between species is 0.25 g biomass L^−1^ water transpired.

During the high-VPD season (Exp. 3), there were significant differences in the mean TE across species among the soils, of the order of 2 g biomass L^−1^ water transpired (Fig. 4A), with the value being highest in the Vertisol, lowest in the sandy soil, and intermediate and similar in the Alfisol and the loamy sand soil. By contrast, during the low-VPD season (Exp. 4), there was no significant variation in mean TE among the different soils, and all were in the narrow range of 3.0–3.3 g biomass L^−1^ water transpired (Fig. 4B). Interestingly, when looking at the species level, TE in pearl millet did not vary among the soils in the high-VPD season (Fig. 5A), whereas in maize it varied considerably with soil type, with the highest values in the Vertisol and Alfisol and the lowest in the sandy soil. For sorghum, significant variation was limited to the Vertisol, where the highest TE value was observed. During the low-VPD season, there was no soil effect on the TE of each individual species, except for a high value in the Vertisol for pearl millet (Fig. 5B).

**Fig. 6.**
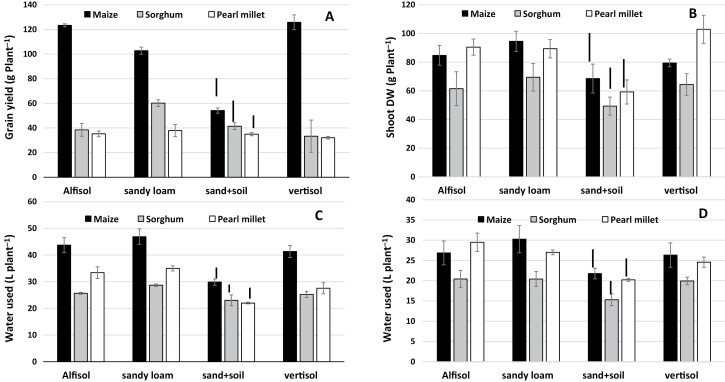
Grain yield, shoot dry weight, and total water used in maize, sorghum, and pearl millet grown in different soil types in lysimeters in the field under well-watered conditions. (A, C) Experiment 3 with relatively high vapor pressure deficit (VPD) and (B, D) Experiment 4 with relatively low VPD (Fig. 1). (A) Grain yield, (B) shoot dry weight, and (C, D) total water used. Data are means (±SE) of three genotypes in each species (Table 1), with five replicate plants being sampled to determine a mean value for each genotype. Bars indicating the LSD at *P*<0.01 between the soil treatments within a species are shown. The LSDs to compare differences between species within each soil are as follows: (A) 6.6 g plant^−1^, (B) 9.0 g plant^−1^, (C) 1.8 L plant^−1^, and (D) 2.1 L plant^−1^.

Grain yield was again much higher in maize than in sorghum and pearl millet during the high-VPD season (Fig. 6A), whilst in the low-VPD season the shoot biomass of maize was only greater than that in sorghum (Fig. 6B).Variations in grain yield across the soils during the high-VPD season were consistent with those found in TE, with maize showing large yield variations and the lowest value in the sandy soil and highest values in the Vertisol and Alfisol (Fig. 6A). By contrast, grain yield hardly varied across the soils in sorghum and did not vary in pearl millet. During the low-VPD season there were hardly any significant variations in shoot biomass across soils in maize and sorghum, although both had slightly lower values in the sandy soil, and the only significant difference was a lower value in pearl millet in the sandy soil (Fig. 6B). Total water used generally followed the trend of variations in TE (Fig. 6C, D). During the high-VPD season, all three species had their lowest water use in the sandy soil, and this was particularly apparent in maize (Fig. 6C). During the low-VPD season, the variations in water use within the species were limited, but for all three they were lower in the sandy soil (Fig. 6D).

### Source–sink effects on TE and water use

**Fig. 7.**
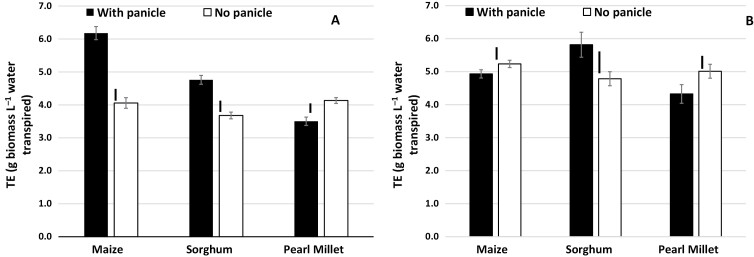
Effects of altering the source–sink balance on transpiration efficiency (TE) for maize, sorghum, and pearl millet grown in lysimeters in the field with or without water stress (Exp. 5). Maize cobs and the panicles in sorghum and pearl millet were removed and the plants were grown under either (A) well-watered conditions or (B) under water stress, in which watering was stopped at 59 d after sowing. (For ease of presentation, ‘panicle’ in the figure also refers to ‘cobs’.) Data are means (±SE) of 10 genotypes for maize and pearl millet, and 16 genotypes of sorghum, with five replicate plants being sampled to determine the mean for each genotype. Bars indicating the LSD at *P*<0.01 for the treatment effect within each species are shown.

Removal of panicles/cobs had significant but contrasting effects on TE among the species. Under well-watered (WW) conditions, removal of maize cobs decreased TE by more than 2 g biomass L^–1^ water, which was ~35% lower than its value in intact plants (Fig. 7A). There was also a significant decrease in TE is sorghum when the panicles were removed, although to a lesser extent than in maize (~1 g biomass L^–1^ water, ~20% decrease). By contrast, pearl millet showed a modest increase in TE when the panicles were removed (~0.5 g biomass L^–1^ water). Similar results for sorghum and pearl millet were found under water-stress (WS) conditions, whereas removal of cobs had no effect in maize (Fig. 7B).

**Fig. 8.**
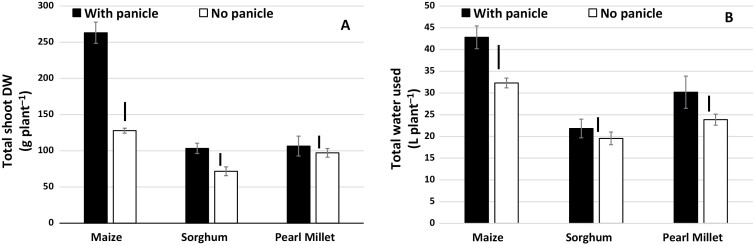
Effects of altering the source–sink balance on shoot dry weight and on total water used for maize, sorghum, and pearl millet grown in lysimeters in the field (Exp. 5) under WW conditions. The source–sink balance was altered by removal of the cobs in maize and the panicles in sorghum and pearl millet (for ease of presentation, ‘panicle’ in the figure also refers to ‘cobs’). (A) Total shoot dry weight at final harvest and (B) total water used. Data are means (±SE) of 10 genotypes for maize and pearl millet, and 16 genotypes of sorghum, with five replicate plants being sampled to determine the mean for each genotype. Bars indicating the LSD at *P*<0.01 for the treatment effect within each species are shown.

Removal of the cobs had a dramatic effect on total shoot biomass in maize, resulting in a decrease of more than 50% under WW conditions (Fig. 8A). By contrast, a decrease of only ~25% was found in sorghum and there was no effect in pearl millet. Under WS conditions, the trend was similar in maize, and there were no differences in shoot biomass in sorghum and pearl millet (Supplementary Fig. S2A). Despite the dramatic decrease in biomass in maize under WW conditions, the total water used was only decreased by ~25% when the cobs were removed (Fig. 8B). Meanwhile, the water used was fairly similar between the two treatments in sorghum and pearl millet. Thus, the water usage of maize did not decrease as much as its biomass did under WW conditions, hence accounting for the significant decrease in TE upon removal of the cobs (Fig. 7A). The trend was similar under water stress in sorghum and pearl millet, but different in maize where the decrease in biomass was about proportional to the decrease in water use (Supplementary Fig. S2) so that TE was similar with and without cobs (Fig. 7B).

## Discussion

### Species differences in TE

Overall, maize had higher transpiration efficiency (TE, i.e. total biomass divided by water transpired) than sorghum and pearl millet (Fig. 2). It was notable that differences in TE between maize and sorghum were only observed when the plants were grown in the Alfisol soil (in which Exps 1 and 2 were conducted), and to a lesser extent in the sandy soil (Fig. 5). These were also the experiments in which the full range of genotypes was included. We have previously used a similar lysimetric system to measure plant water use throughout the cropping cycle and to determine TE in sorghum (Vadez *et al.*, 2011b), pearl millet (Vadez *et al.*, 2013a), and groundnut (Vadez and Ratnakumar, 2016), and to obtain agronomically relevant evaluations of yield (Vadez *et al.*, 2014). Therefore we are confident of the robustness of the results obtained in this current study. Despite its importance as a factor for yield enhancement in water-limited environments, there have been few studies that have compared TE across C_4_ species, with only five reports that make side-by-side comparisons between maize, sorghum, and pearl millet or finger millet (Muchow, 1989*a*, 1989*b*; Singh and Singh, 1995; Rurinda *et al.*, 2014; Schittenhelm and Schroetter, 2014; van Oosterom *et al.*, 2021), three of which have assessed water-use efficiency (WUE). Across the wider literature, the results reported tend to conclude that maize is superior in terms of yield performance (e.g. Farré and Faci, 2006), even under fairly limited water availability (Rurinda *et al.*, 2014), which is in agreement with our data (Figs 3, 6). With regards to WUE, Singh and Singh (1995) and Farré and Faci (2006) have reported a similar range of values for maize, sorghum, and pearl millet, with a slight advantage for sorghum under water stress. Zegada-Lizarazu *et al.* (2012) found that sweet sorghum had higher WUE than maize under water deficit, and similar results were reported by Bhattarai *et al.* (2020) and Roby *et al.* (2017). However, few genotypes were used in these studies compared to the 36 that we used. Some of our sorghum genotypes were comparable with some maize genotypes, suggesting that comparison needs to include a wide range of genotypes to represent the species. Only one recent study has used a large range of genotypes, namely eight maize hybrids and 21 2dwarf and 3dwarf sorghum genotypes, and no differences in TE were found between the two species (van Oosterom *et al.*, 2021). Unfortunately, the soil type used in this study was not mentioned, but we speculate that it could have been similar to the Vertisol we used, as the experiment was located in Gatton, Queensland, Australia, where this soil type predominates. If so, this would agree with the absence of a difference in TE between maize and sorghum that we also found in Vertisol (Fig. 5). Overall, our results most clearly distinguished maize and pearl millet in terms of differences in TE, and better TE in maize compared with sorghum appeared to be dependent on soil type, being present in the Alfisol and sandy soil but not in the Vertisol and the sandy loam. Additional data would be required to take into account possible differences in root:shoot ratios among species and their effects on the calculated TE values.

Aside from the possible soil effects in the sorghum versus maize comparison, there are several interpretations of the differences in TE that we found between the species. One explanation could be that there are differences between the species in the capacity to restrict transpiration under high VPD that would lead to differences in TE (see Vadez *et al.*, 2014 for a review). Indeed, a recent study investigating water-conserving traits in the same genotypes of maize, sorghum, and pearl millet that we used showed that all three species were able to restrict transpiration under such conditions, although to a much larger extent in maize than in sorghum and pearl millet (Choudhary *et al.*, 2020). Since most of the pearl millet and sorghum material used in our study lacked transpiration sensitivity to high VPD compared to the maize genotypes, they would have lower TE, as previously hypothesized by Sinclair *et al.* (2005). In fact, many of the elite varieties of sorghum and pearl millet used in these experiments were bred for the rainy season environment, i.e. targeting seasons with predominantly low VPD conditions, and hence the ability to restrict transpiration has not been selected for as an adaptive trait. This could explain the large differences in TE that we found, at least in high-VPD seasons (e.g. Fig. 5), and the differences between our results and those of van Oosterom *et al.*, (2021), who most likely used sorghum genotypes bred for the severe stress conditions of Australia.

A second explanation is the breeding history. Maize has been actively bred for about a century, whereas breeding for sorghum and pearl millet is a lot more recent (from the 1970s) and has not received the same financial and research support (Bänziger *et al.*, 2006). An increase in plant growth rate over time is one of the factors that explains the yield increases in maize, which could be related to sustained photosynthetic rates (Hammer *et al.*, 2009), and which could also have driven up TE. We therefore hypothesize that an increased sink strength in modern maize varieties such as those used in our study could have increased photosynthesis and consequently driven down the *C*_i_/*C*_a_ ratio in the leaves, thereby increasing TE according to Condon *et al.* [2002; TE=0.6*C*_a_(1–*C*_i_/*C*_a_)/(*W*_i_–*W*_a_)]. This could explain why we found differences in TE among the species, especially in the low-VPD season when no restriction of transpiration would have been expected (e.g. Fig. 2).

A third explanation could be that there are differences in biomass partitioning, as suggested recently by Velázquez *et al.* (2017), whereby sorghum and pearl millet could have greater allocation to the roots than maize. We did not extract roots and hence they were not included in our measurements of biomass. Root:shoot ratios appear to vary in maize and sorghum, from values up to 0.1 in maize (Ordóñez *et al.*, 2020) to values of ~0.3 in sorghum (van Oosterom *et al.*, 2011). Applying these values to adjust the biomass results in our Exps 1 and 2 removes the differences between maize and sorghum under well-watered conditions but not under water stress, where maize maintains a higher TE.

Overall, our results showed that maize had superior TE compared to pearl millet, and also compared to sorghum at least in Alfisol and sandy soils, and especially under water stress, and that this had direct effects on yield. A wider range of TE values was found in sorghum and pearl millet than in maize, and this provides scope for breeding for higher TE in these two species, which their C_4_ physiology theoretically allows. Improving the capacity for restriction of transpiration would be one approach toward this.

### Soil effects on TE

Soil type had an effect on TE (Fig. 4), and this was species-dependent (Fig. 5). Very few studies have looked at the effect of soil on plant productivity in relation to water. Razzaghi *et al.* (2012) found no differences in WUE among soils and Bello *et al.* (2019) found only very small differences between two sandy soils. However, a simulation study has shown that maize productivity is sensitive to soil hydraulic properties, and that water-related productivity would be affected differently by climate change in different soils (Pinheiro *et al.*, 2019). We have two interpretations for the differences in TE among the different soils that we used, and we build upon the hypothesis of a link between high TE and the capacity to restrict transpiration under high VPD (Sinclair *et al.*, 2005; Vadez *et al.*, 2014).

 Our first interpretation is that differences in matric potential between the different soils could have altered the transpiration response to the high-VPD conditions. Soil texture, where the fractions of clay, silt, and sand particles are important factors, is considered to be the main driver behind the water retention properties of soils (Zhuang *et al.*, 2001; Scharwies and Dinneny, 2019). The sandy soil and the Vertisol would be expected to possess opposite properties. Higher matric potential in the sandy soil, and to a certain extent in the Alfisol, would have allowed the plants to take up water relatively quickly from the soil profile to support the high transpiration demand in the high-VPD season, thus leading to lower TE. In contrast higher water retention in a soil with lower matric potential, as in the case of the high clay content of the Vertisol (50%), could have induced a restriction to transpiration, leading to higher TE. This interpretation is in agreement with the results of a study by Yang *et al.* (2019) who used super-absorbent polymers to increase water retention in a sandy soil, which resulted in an increase in WUE in maize and also in an increase in root density that allowed better access of the roots to water. The fact that we observed no soil effect on TE in the low-VPD season also favors this interpretation (Fig. 4). The location of water retention in the soil remains unclear. Newman (1969) considered that the resistance of the rhizosphere to water movement towards the roots is relatively unimportant and that the resistance of the soil beyond the roots (pararhizal resistance) could be a limiting factor even at water content values close to field capacity. However, other studies have shown that the connection of the roots to the soil through the rhizosphere is a critical factor (Sperry *et al.*, 1998; Carminati *et al.*, 2017; Ahmed *et al.*, 2018). The thin layer of the rhizosphere has the potential to disrupt the connection between the soil and the plant and to create a situation in which water movement toward the roots is reduced, thus triggering stomatal closure to maintain the hydraulic integrity of the plant (Carminati *et al.*, 2016). Carminati and Javaux (2020) have recently argued that a better understanding of the hydraulic connection between the plant and the soil is essential to fully understand responses to drought, especially under progressive drying. The authors state that the magnitude of the drop in water potential between the bulk soil and the soil–root interface increases dramatically at different levels of dryness for different soil types. This could have happened in our lysimeters, which were only watered once a week and thus would have experienced cycles of drying and re-wetting even though water stress *per se* was avoided. The production of mucilage in response to drought to maintain the hydraulic connection between the roots and the soil would be an important consideration (Ahmadi *et al.*, 2017; Ahmed *et al.*, 2018). Root hairs also appear to be an active part of this hydraulic connection. For instance, in barley mutants without root hairs, whereas no differences were detected under well-watered conditions, the plant response to drought was more rapid in the mutants than in the wild-type (Carminati *et al.*, 2017). The hypothesis of mucilage and/or root hairs having an effect on the transpiration response under high-VPD conditions has not been examined and presents an interesting avenue for future research.

These putative differences in soil hydraulics, however, are not enough to explain the differences in TE that we observed between the species, since interactions with soil type were apparent (Fig. 5). Hence our second interpretation involves differences in root hydraulic conductance among the three species interacting with the soil type to affect the way the plants responded to increased evaporative demand. Root density, distribution in the soil volume, conductivity, and anatomy are all traits that could potentially determine overall hydraulic conductance (Ahmed *et al.*, 2018), and all might show differences between species and between soil types. The root to shoot area ratio could also be an important factor in putative species differences because rhizosphere conductance becomes limiting for water absorption below certain threshold ratios, and these depend on the soil (Sperry *et al.*, 1998). The fact that TE under high-VPD conditions was the same in pearl millet in the sandy soil as in the Vertisol, and was at the same level as maize in the sandy soil (Fig. 5A), suggests that there were differences in root hydraulic conductance between the two species in the Vertisol. Taken together with the difference between maize and sorghum in the Alfisol, the results suggest higher hydraulic conductance in pearl millet and sorghum than in maize, thus allowing them to support transpiration under high evaporative demand in these types of soil. We did not examine the root structure and so we do not know whether the roots of pearl millet occupied the soil volume in a different way to maize in the Vertisol, thereby allowing the hydraulic connection between the roots and the soil to be maintained. A recent study has indeed suggested that pearl millet has a dense and profuse root system that allows a small gradient of water potential to be maintained even as the soil dries (Cai *et al.*, 2020). These results also imply that the fitness of crop species grown in a high-VPD environment will be soil type-specific. For instance, maize did not perform well in the sandy soil under such conditions, whereas pearl millet was suited to all the soil types.

In summary, whilst there is a lot of published work on soil and root hydraulics, it is only recently that they have been studied in conjunction. Examining their interactions has great potential for explaining differences in TE between species. In terms of the shoots, it would be interesting to determine whether soils of different matric potentials are able to trigger different transpiration–VPD response curves, whether and how much these response curves are species-dependent, and how these responses depend on the soil water content. In terms of the roots, there is a crucial lack of data on the traits that contribute to hydraulic conductance in crop species, on their interactions with soil texture, on root-to-shoot area ratios, and on how any differences in these factors will affect the transpiration–VPD response curves. Future research that examines both soil and root variations and their interactions is needed for us to have a more complete picture of of the restrictions on transpiration that occur under high VPD.

### Source–sink effects on TE

Under well-watered conditions there was a strong reduction in TE in maize as a result of removal of cobs, whilst removal of panicles resulted in a significant but smaller reduction in sorghum and had no effect in pearl millet (Fig. 7). A caveat should be noted for these TE data because the roots were not taken into account in the calculation, and they could have become a sink for carbon in the absence of grain on the shoots. In four sorghum hybrids that were exposed to water stress, the root:shoot biomass ratio has been shown to increase dramatically from ~0.3 in plants with normal seed set to ~0.7 in plants with no seed set (van Oosterom *et al.*, 2011). However, a similar effect for maize in our study is very unlikely because mean shoot biomass decreased from 263 g plant^−1^ to 127 g plant^−1^. Assuming that all the loss in shoot biomass (~130 g) was gained by the roots, then it would imply a root:shoot ratio >1, which is drastically outside the typical range of 0.04–0.13 that has been found across a variety of different environments in the field (Ordóñez *et al.*, 2020), and much higher even than the root:shoot ratio in sorghum with no seed set (van Oosterom *et al.*, 2011). Therefore, removal of the cobs depressed biomass accumulation in the high-VPD season, but did not decrease transpiration to a similar extent. Our interpretation is that regulation of transpiration under high VPD, which is an active water conservation trait in maize (Choudhary *et al.*, 2020), could have been lost as a result of the loss in sink capacity. Indeed, loss of the ability to regulate stomata has been reported following de-fruiting in apple at late stages in development, with WUE increasing when fruit are removed during the summer, but decreasing when they are removed at maturity, putatively because photosynthate then goes to other perennial sinks in the shoots and roots in the summer (Wibbe and Blanke, 1995). The reason for the maintenance of a high transpiration rate in maize is unclear and it could relate in part to the need to dissipate light energy by photorespiration (Kozaki and Takeba, 1996; Busch, 2020). Decreasing sink capacity usually leads to an inhibition of photosynthesis (Ainsworth and Bush, 2011), which is triggered by stomatal closure following the sensing of excess glucose levels by hexokinase (Kelly *et al.*, 2014); however, this coordination is not always tight. For instance, compared to the wild-type, photosynthetic rate can be depressed by ~75% in transgenic lines with a lower Rubisco content without there being any change in stomatal opening (von Caemmerer *et al.*, 2004). Similarly, the photosynthetic rate of ageing leaves declines while transpiration is maintained or declines to a lesser extent, resulting in a lower intrinsic WUE at the leaf level (Atkinson *et al.*, 1989). This could have also been the case in our maize plants, where high transpiration under high VPD would have led to decreased TE whilst accumulation of biomass was depressed in the absence of a sink. By contrast, sorghum and particularly pearl millet kept producing tillers (including nodal tillers in the case of pearl millet), and thus unlike maize might never have had real sink limitation.

There has been very limited work addressing the relationship that TE or WUE has with sink capacity in crops, and more specifically with source–sink relationships and their role in the regulation of photosynthesis and/or stomatal conductance. Several studies have shown links between TE and biomass allocation that reflect source–sink relationships. For instance, Zegada-Lizarazu *et al.* (2012) found that TE was high and similar to maize in a sweet sorghum line, which could be a consequence of the sucrose sink in the sorghum stem acting on photosynthetic efficiency. Similarly, TE has been shown to be high and comparable with maize in forage sorghum lines (Roby *et al.*, 2017; Bhattarai *et al.*, 2020), which could be the result of less leaf area supporting a higher biomass sink, leading to a higher TE (Condon *et al.*, 2002). In sunflower, TE is positively associated with a decrease in the biomass allocation to leaf area, and to a decrease in the leaf area to biomass ratio (Velázquez *et al.*, 2017). Similarly, in wheat and groundnut, TE has been shown to be positively related to an increased biomass allocation to the stem (Masle and Farquhar, 1988; Puangbut *et al.*, 2009).

In summary, our results suggest that source–sink balance is likely to affect TE. Decades of breeding for stronger sink strength in maize will, at least in part, have given it an advantage over less-bred crops such as pearl millet and sorghum. The interpretation of differences in TE from the perspective of sink strength and source–sink balance provides a potentially interesting avenue to explore for further improvements in TE. This also involves aspects of biomass allocation between the roots and shoot, which in turn could have interactions with and implications for the soil–plant hydraulic balance.

### Concluding remarks

Factors other than the restriction of transpiration under high VPD can alter TE in a crop-dependent manner, namely the soil type and the source–sink balance. Despite having a physiologically similar photosynthetic apparatus, maize had a consistent advantage in terms of TE over pearl millet in all soil types, , although this was less under water stress, and over sorghum when grown in an Alfisol. This was related in part to its higher capacity to restrict transpiration under high VPD in the set of genotypes that we examined, but also to the fact that its breeding history has possibly altered the source–sink balance and/or photosynthetic efficiency. The soil type—here interpreted as the soil hydraulic characteristics—had a strong influence on TE, with higher values being observed in the high-clay Vertisol, although this was species-dependent since TE varied among the soil types in maize but not in pearl millet. Our interpretation is that soil-specific differences in root traits exist among the species that alter the soil–plant hydraulic continuum in ways such that transpiration under high VPD is eventually restricted differently among the species, thus ultimately resulting in differences in TE that are soil-specific. Our results highlight the need for a lot more data on soil effects on the restriction of transpiration, on species differences in soil-specific root traits that are relevant hydraulic conductance, and on root-to-leaf area ratios, all of which determine the hydraulic limits of the rhizosphere conductance in a soil-dependent manner. We found that source–sink relationships also had a strong influence on TE, exemplified by the fact that removal of cobs in maize dramatically depressed TE while no effect was observed when panicles were removed in pearl millet. This provides scope to further improve TE by harnessing the characteristics of the source–sink balance that contribute to increasing it, for instance focusing on the leaf-to-biomass ratio or the sink strength.

## Supplementary data

The following supplementary data are available at *JXB* online.

Fig. S1. Total water extracted in maize, sorghum, and pearl millet in Exps 1 and 2.

Fig. S2. Total shoot dry weight and total water used in Exp. 5.

erab251_suppl_Supplementary_Figures_S1-S2

## Data Availability

The data supporting the findings of this study are available from the corresponding author, Vincent Vadez, upon request.
