# Supplementary material for: Transpiration efficiency: insights from comparisons of C4 cereal species
Source: J Exp Bot. 2021 Jun 3;72(14):5221–34. doi: 10.1093/jxb/erab251 (PMC8272567; doi:10.1093/jxb/erab251)

Supplementary Figure 1. Total water extracted (A, B) in maize, sorghum, and pearl millet, measured in lysimeters (Vadez et al., 2014) in Experiment 1 (A, C) and Experiment 2 (B, D), in several water regimes, i.e. well watered (WW), or water stress imposed at the time of flowering of each crop in Experiment 1 (WS), or imposed at the time of pearl millet flowering (WS3), sorghum flowering (WS2) or maize male flowering (WS1) in Experiment 2. Data are average (+/- SE) of genotype means within species (n = 10 for maize, n = 16 for sorghum, n = 10 for pearl millet). Genotypic mean data were the average of five replicated lysimeters per genotype.

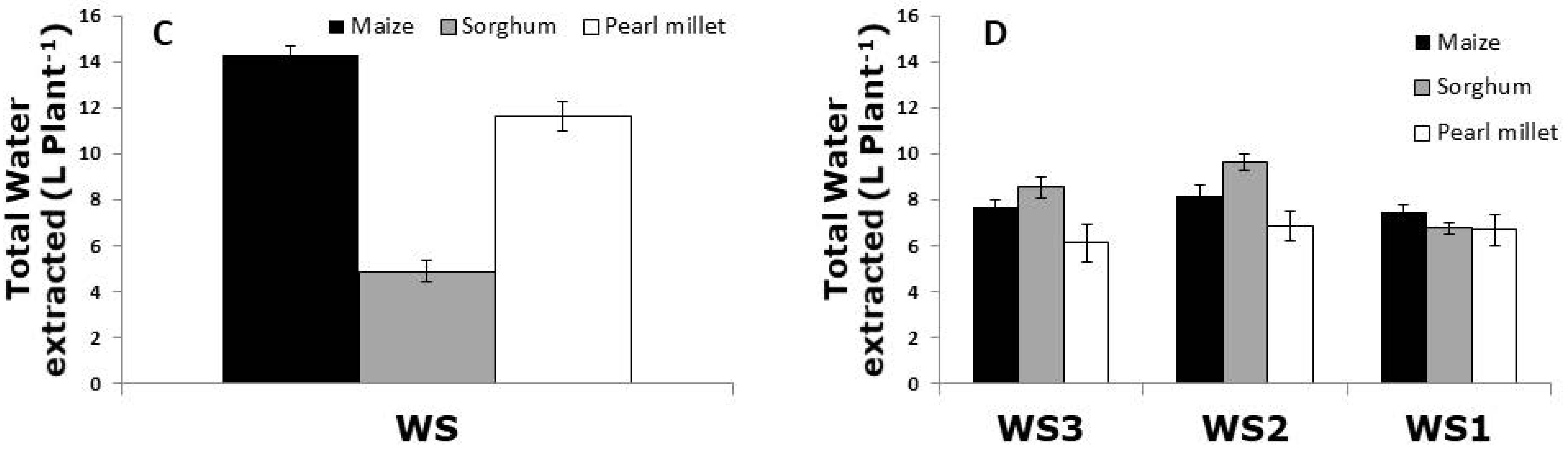

Supplementary Figure 2. Total dry weight (DW) (A) and total water used (B) measured in lysimeters (Vadez et al., 2014), assessed in maize, sorghum and pearl millet, under water stressed conditions, where reproductive organs (female organ in maize) were either removed (no panicle) or left untouched (with panicle). Data are average (+/- SE) of genotype means within species (n = 10 for maize, n = 16 for sorghum, n = 10 for pearl millet). Genotype means were the average of five replicated lysimeters per genotype.

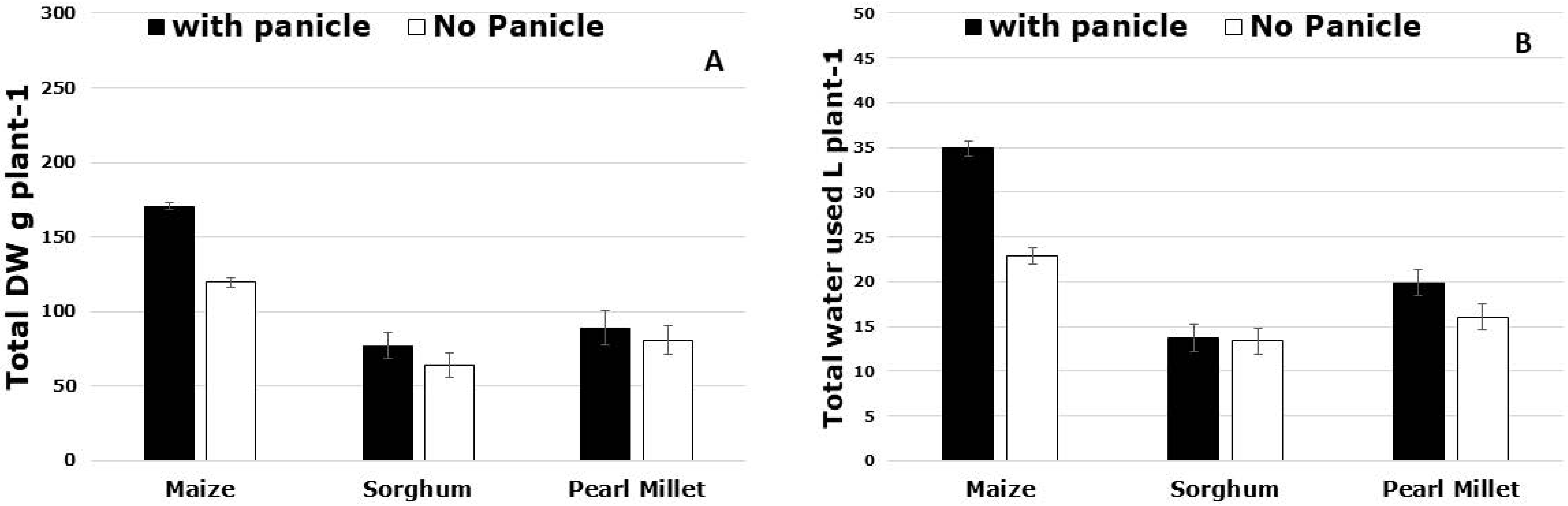

Supplement: erab251_suppl_Supplementary_Figures_S1-S2 [file erab251_suppl_supplementary_figures_s1-s2.pdf]
